# Prevalence and risk factors for recurrent *Staphylococcus aureus* small-colony variants in people with cystic fibrosis followed at the Tuscan Regional Reference Center

**DOI:** 10.1007/s10096-025-05313-3

**Published:** 2025-10-30

**Authors:** Vito Terlizzi, Cristina Fevola, Daniela Dolce, Silvia Campana, Angelica Terri, Giovanni Taccetti, Elena Chiappini

**Affiliations:** 1 Department of Pediatric Medicine, Meyer Children’s Hospital, IRCCS Cystic Fibrosis Regional Reference Centre, Florence, Viale Gaetano Pieraccini 24, Florence, 50139 Italy; 2 Department of Health Sciences, University of Florence, Meyer Children’s Hospital IRCCS, Viale Gaetano Pieraccini 24, Florence, 50139 Italy; 3 Infectious Diseases Unit, Meyer Children’s Hospital IRCCS, Florence, Viale Gaetano Pieraccini 24, Florence, 50139 Italy

**Keywords:** Small colony variants, Staphylococcus aureus, Trimethoprim-sulfamethoxazole, Elexacaftor/tezacaftor/ivacaftor, Outcomes

## Abstract

**Purpose:**

This study aimed to determine the prevalence of *Staphylococcus aureus* small colony variants (SCV) in people with cystic fibrosis (pwCF), evaluate the clinical differences of single versus multiple detections of SCVs in respiratory cultures, and assess antibiotic resistance.

**Methods:**

This monocentric retrospective study included pwCF colonised by *S. aureus* SCVs between January 1, 2017, and December 31, 2023, at the CF centre of Florence, Italy. Clinical data were collected, and patients with single versus recurrent SCV detections were compared to identify risk factors for recurrent SCVs.

**Results:**

Among 154 pwCF (62 children, 92 adults), SCV was detected in 38.31%. Univariate analysis identified lower percent predicted forced expiratory volume in the first second (ppFEV_1_) (OR: 0.49, *p* = 0.04), prior trimethoprim-sulfamethoxazole (TMP-SMX) use (OR: 2.639, *p* = 0.005), chronic azithromycin (OR: 2.228, *p* = 0.020), and chronic/intermittent *Pseudomonas aeruginosa* colonisation (OR: 2.107, *p* = 0.049) as risk factors for recurrent SCVs. In multivariate analysis, prior TMP-SMX use was the sole independent risk factor for recurrent SCVs (OR: 2.280, *p* = 0.028). A significant decline in respiratory function over time (*p* = 0.025) was observed in patients with recurrent SCVs, but not in those with a single SCV episode. Resistance to TMP-SMX was observed in nearly all isolates tested (231/234) with available antibiotic susceptibility testing results. SCV detection frequency decreased over the study period (19.06% in 2017 vs. 5.83% in 2023, *p* < 0.00001), probably due to the increased use of elexacaftor-tezaxaftor-ivacaftor.

**Conclusions:**

*S. aureus* SCVs were highly prevalent in pwCF, although their frequency declined over time with the widespread use of elexacaftor–tezacaftor–ivacaftor. Recurrent SCV detections, but not single episodes, were associated with a progressive decline in respiratory function. Prior TMP-SMX exposure was identified as an independent risk factor for recurrent SCV detections. The clinical implications of these findings require further investigation to clarify causality and guide management strategies.

**Supplementary Information:**

The online version contains supplementary material available at 10.1007/s10096-025-05313-3.

## Introduction

 Cystic fibrosis (CF) remains the most common life-threatening genetic disorder among Caucasian populations [1, 2], with an incidence of approximately 1 in 2,500 live births in Italy [3]. Pulmonary involvement represents the primary clinical challenge in people with CF (pwCF). Thickened mucus predisposes pwCF to bacterial respiratory infections [1], leading to progressive lung damage, chronic obstructive pulmonary disease, chronic respiratory failure, and the need for repeated antibiotic courses [4].

The advent of CF transmembrane conductance regulator (CFTR) modulators has revolutionized the clinical management of CF. These drugs can enhance CFTR protein function (potentiators, such as ivacaftor) or correct protein defects (correctors, such as elexacaftor, lumacaftor and tezacaftor). Combined potentiator and corrector therapies have demonstrated significant benefits in pwCF with specific variants, such as F508del [5–8]. Notably, elexacaftor/tezacaftor/ivacaftor (ETI) has been shown to have substantial clinical efficacy, altering the disease trajectory and resulting in significant improvements in lung function, reduced pulmonary exacerbations, decreased sweat chloride levels, fewer hospitalizations, increased body mass index (BMI), enhanced quality of life, and reduced infection rates and mortality [7, 9]. *Staphylococcus aureus* is the most frequently retrieved pathogen from respiratory samples, followed by *Pseudomonas aeruginos*a (Pa) [3]. Methicillin-susceptible *S. aureus* (MSSA) is more prevalent in younger pwCF (< 15 years), while the incidence of methicillin-resistant *S. aureus* (MRSA) increases between 10 and 30 years of age [3, 10].

Recently, the detection and clinical impact of *S. aureus* small colony variants (SCVs) have garnered increasing attention. SCVs exhibit a distinct phenotypic profile, characterized by reduced colony size, slow growth, absence of carotenoid pigment, diminished hemolytic activity, and coagulase negativity. SCVs exhibit variable phenotypic stability and are often associated with attenuated virulence due to reduced expression of virulence factors and alterations in metabolic pathways [11].

These variants arise from adaptations to adverse environmental conditions (e.g., low pH, oxidative stress, nutrient deficiency, and antibiotic exposure), leading to mutations that reduce virulence factor expression [12]. This adaptation enables bacterial survival within the host and resistance to antimicrobial agents, resulting in chronic infection [12]. Studies have shown that SCVs are more frequently detected in patients with advanced lung disease, raising two possibilities: (1) the lower respiratory function observed in SCV-colonized pwCF may be a direct consequence of the infection; and (2) pwCF with more advanced disease may be more susceptible to SCV detection [13]. Given that SCVs detection frequently occurs in patients diagnosed with CF at a young age, it is plausible that prior exposure to multiple courses of antibiotic therapy contributes to the emergence of SCV infections.

Other factors that may influence SCVs emergence have also been investigated. While dornase alfa may be associated with increased SCV detection, hypertonic saline appears to reduce SCV infection [13]. Colonization with Pa may also increase the risk of SCVs emergence [13].

Although several studies have explored SCVs detection and impact in both CF and non-CF populations, the literature remains inconsistent and often relies on small sample sizes [14, 15]. Risk factors for SCVs detection are not well-defined, and clear management guidelines for SCV-colonized pwCF are lacking. Furthermore, the impact of CFTR modulator therapy on SCVs detection and clinical outcomes remains unclear [16]. Addressing these knowledge gaps is essential for optimizing the management of pwCF, including the development of strategies to prevent SCV colonization and to guide appropriate antibiotic treatment upon SCV isolation. The aims of the study were:

1. To retrospectively assess the detection of *S. aureus* SCVs between 2017 and 2023 in pwCF attending the Regional Reference Center for Cystic Fibrosis of the Meyer University Hospital IRCCS.
2. To evaluate clinical differences in pwCF with single versus multiple detections of SCVs during the study, considering percent predicted forced expiratory volume in 1 s (ppFEV1), BMI, and the incidence of respiratory exacerbations.
3. To assess the effect of ETI on SCV epidemiology in the cohort, with specific attention to detection rates and persistence (likelihood of multiple detections) over time.
4. To evaluate the proportion of antibiotic resistance among SCV isolates, particularly to trimethoprim–sulfamethoxazole (TMP–SMX) and other antibiotics.

## Methods

### Study design and population

This retrospective, single-center study was conducted at the Regional Reference Center for Cystic Fibrosis at the Meyer University Hospital IRCCS. Ethical approval was obtained from the local ethics committee (approval number 50/2024, dated April 19th, 2024). Informed consent was obtained from all adult pwCF or their legal guardians for minors. The study included all pwCF of any age who had at least one detection of *S. aureus* SCV between January 1, 2017, and December 31, 2023. Inclusion criteria were a confirmed diagnosis of CF based on established diagnostic guidelines [17] and clinical follow-up at least quarterly, according to standard of care [18].

### Data collection

For each patient, demographic information and CF diagnosis data were collected. The dates of the first and subsequent detections of *S. aureus* SCV were recorded, along with the detection source (sample type), co-occurring pathogens, antibiotic resistance profiles, and the patients’ health status. Clinical data for each patient, both before and after *S. aureus* SCV detection, are presented in Supplementary Table A. For patients aged 6 years or older, at least two measures of ppFEV1 were obtained, expressed as a percentage of the predicted value for age according to standardized reference equations for spirometry [16].

The detection of SCV more than once during the study period was defined as “recurrent” SCV detection.

Respiratory tract microorganisms were identified from throat swabs and sputum samples (collected from patients able to expectorate). Chronic Pa infection was defined according to the modified Leeds criteria [19]. Pulmonary exacerbations were defined according to Flume et al. [20].

According to Italian legislative guidelines, the prescription of ETI was allowed since October 2019 for adult pwCF with advanced lung disease (ppFEV1 < 40%) and subsequently approved for pwCF aged 12 and above (July 2021) and 6 and above (September 2022).

### Laboratory methods

*S. aureus* was cultured on mannitol salt agar (MSA) plates (bioMérieux, France), a selective medium. Airway samples were processed according to standard microbiological procedures following guidelines for CF patients (Recommendations SIFC Microbiologists Group) [21]. To ensure detection of slow-growing SCV, incubation was extended to 72 h.

Presumptive identification of *S. aureus* isolates was based on characteristic colony morphology on MSA. Confirmation was achieved using Matrix-Assisted Laser Desorption/Ionization-Time Of Flight Mass Spectrometry (MALDI-TOF) [22]. Despite their atypical, pinpoint colony morphology, S. *aureus* SCVs are correctly identified at the species level as *S. aureus* by MALDI-TOF mass spectrometry. Methicillin resistance was determined using Oxa Screen Test Agar (Becton and Dickinson), a chromogenic medium. Antibiotic Susceptibility Testing (AST) was assessed using the BD Phoenix™ M50 system (Becton Dickinson) with PMIC-96 panels for Gram-positive bacteria. Antimicrobial susceptibility results were interpreted according to the European Committee on Antimicrobial Susceptibility Testing (EUCAST) clinical breakpoints [23]. Due to the unique metabolic characteristics of *S. aureus* SCVs, automated methods cannot reliably evaluate their chemosensitivity [24]. Therefore, AST for these variants was determined manually using the modified disc diffusion method on blood agar plates to support their growth [25].

### Statistical analysis

Continuous variables are presented as mean and standard deviation (SD) or median with interquartile range (IQR), as appropriate. Categorical variables are described as frequencies and percentages. Where applicable, 95% confidence intervals (95% CI) for proportions were calculated using the Wilson method. The χ² test or Fisher’s exact test (for variables with expected cell counts ≤ 5) was used to compare categorical variables. The Mann-Whitney U-test was employed to analyze differences between continuous variables with non-parametric distributions. Linear regression models were utilized to assess the impact of SCV detection on changes in ppFEV_1_, BMI, the number of exacerbations, antibiotic resistance, and to evaluate the effect of ETI on the frequency of SCV detection. Univariate and multivariate logistic regression analyses using the Cox proportional hazards model were conducted to identify potential risk factors for recurrent detection. For these analyses, ppFEV1 values and chronic colonization status were recorded using the measurement closest to and preceding the first SCV detection (within 6 months). Prior TMP-SMX exposure was assessed within the 12 months preceding the first SCV detection.

Statistical significance was defined as a p-value < 0.05. All statistical analyses were performed using IBM Statistical Package for the Social Sciences (SPSS) version 23.0 (IBM Corp, Armonk, NY, USA).

## Results

### Population study

During the study period, 402 pwCF were followed at the CF center of Florence, Italy. Of these, 154 (38.31%, 95% CI: 33.69%−43.15%) had at least one culture positive for *S. aureus* SCV and were included in this study. Patient characteristics are summarized in Supplementary Table B. The cohort included 62 children (40.26%, 95% CI: 32.84%−48.15%) and 92 adults (59.74%, 95% CI: 51.85%−67.16%). The median age of pediatric pwCF was 12.28 years (IQR: 9.30–13.98.30.98), while the median age of adult pwCF was 27.87 (IQR: 22.89–36.70) years. Notably, 76.63% of included patients carried at least one F508del variant.

Among the 154 study pwCF, 73 (47.40%, 95% CI: 39.68%−55.26%) experienced a single SCV detection episode, whereas 81 (52.60%, 95% CI: 44.74%−60.32%) experienced recurrent SCV detections during the study period. In 24 patients, SCV was detected twice, in 16 patients three times, in 6 patients four times, in 9 patients five times, in 6 patients six times, in 7 patients seven times, and in 13 patients at least eight times. The median age at the time of the first SCV detection was 19.23 years (IQR: 12.07–30.14) in pwCF with a single episode, and 22.63 years (IQR: 15.63–29.36) in those with multiple episodes.

### SCV detection and impact of ETI modulator among study population

A total of 446 detections were recorded throughout the study period. A significantly higher percentage of detections occurred in 2017 (χ² = 38.818; *p* < 0.00001) and 2018 (χ² = 103.721; *p* < 0.00001) compared to other years. Specifically, 19.06% (95% CI: 15.68–22.96%, 85/446) of detections occurred in 2017, and 32.51% (95% CI: 28.33–36.99%, 145/446) in 2018. Although a higher frequency of SCV detection was observed in adult patients with respect to children, this difference was not statistically significant (*p* = 0.468) (Supplementary Table C).

**Fig. 1:**
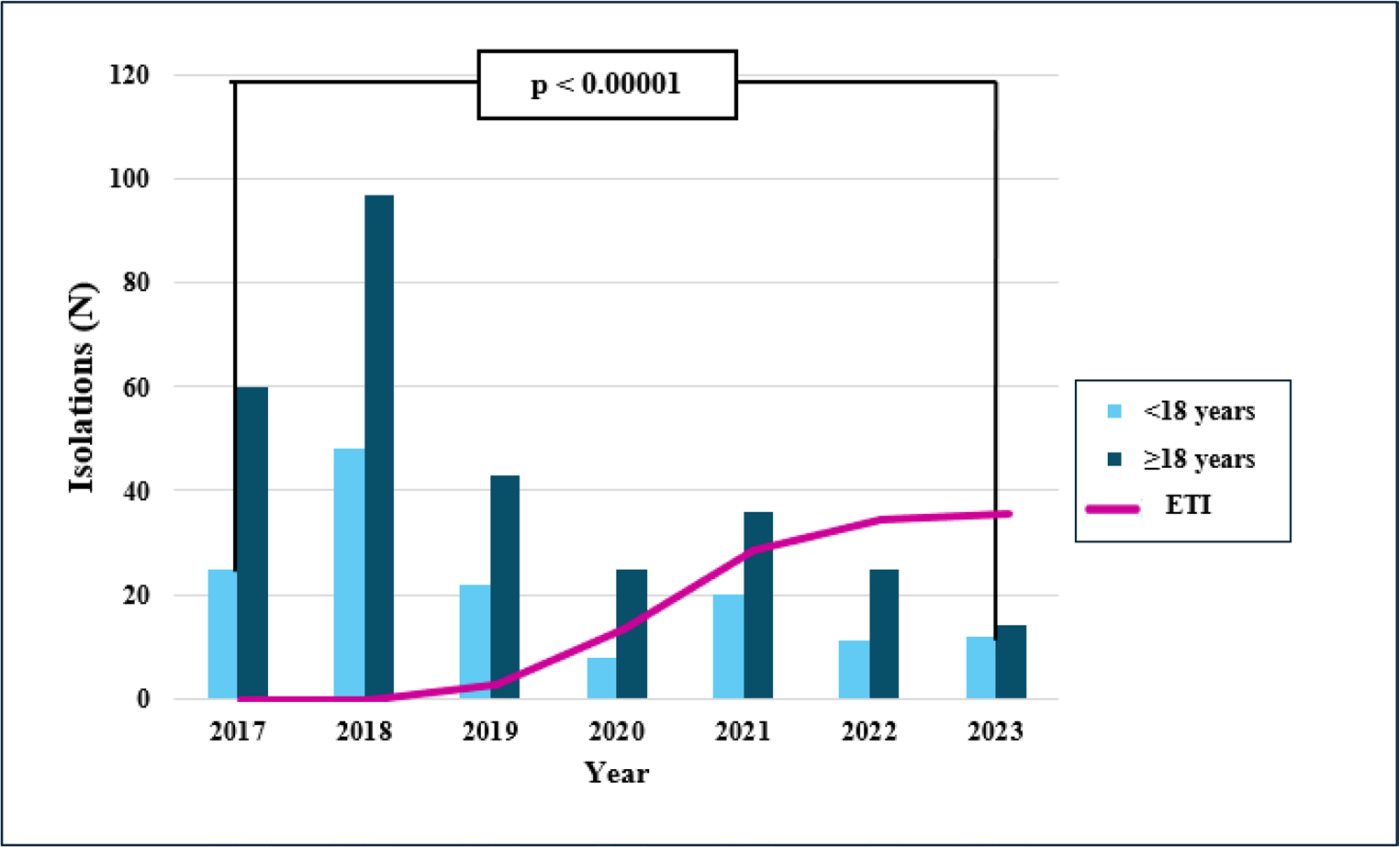
Number of detections over the years, grouped by patient age and correlated to the introduction of the ETI therapy

**Fig. 2:**
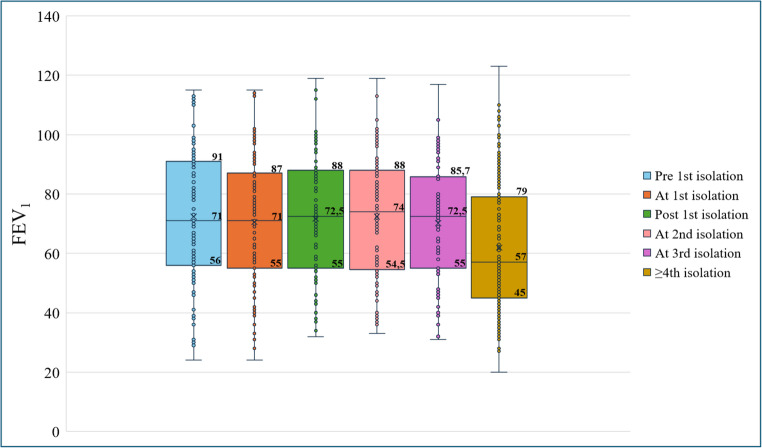
Median and IQR of ppFEV_1_

Furthermore, we analyzed the number of SCV detections in relation to the use of ETI therapy. A substantial decrease in detection episodes was observed between 2018 and 2020 (Fig. 1). Among 446 detections, 115 occurred in pwCF who never initiated ETI therapy (therefore without F508del). Of the remaining 331 detections, only 36 (10.88%, 95% CI: 7.96–14.69%) occurred after the drug was started (Supplementary Table D).(Fig. 2)

### Antibiotic susceptibility testing results

Of the 234 available AST, 98.7% (231/234) showed resistance to TMP-SMX. No significant difference in resistance rates was found between adults and children (*p* = 0.553). However, all the *S. aureus* SCV isolates remained susceptible to vancomycin, linezolid, and teicoplanin.

### Clinical differences in patients with single or recurrent SCV detection

**Fig. 3:**
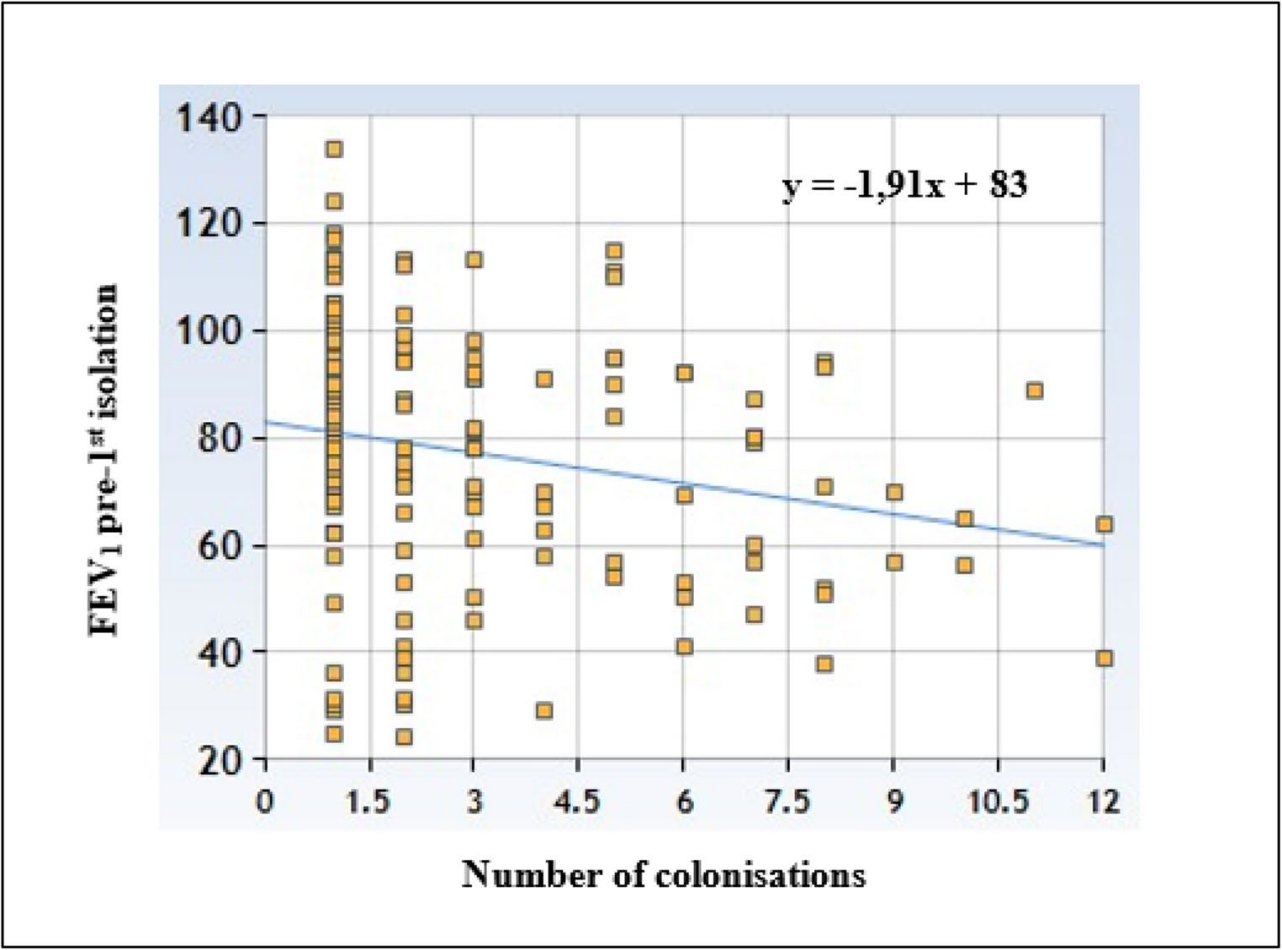
Linear regression analysis between the number of SCV’s isolations and ppFEV_1_ pre-first detection

In pwCF experiencing a single SCV detection, median ppFEV1 values decreased from 85% (IQR: 72–97.5%) before detection to 83.5% (IQR: 70.5–97%) after detection, although this change was not statistically significant (*p* = 0.522). In contrast, in pwCF with recurrent SCV detections, median ppFEV1 values slightly increased from 71% (IQR: 56–91%) before the first detection to 72.5% (IQR: 55–88%) after the first detection, followed by a significant decline to 57% (IQR: 45–79%) after the third detection (z = − 2.238; *p* = 0.025) (Supplementary Table E).Notably, individuals with a single SCV detection displayed higher pre-detection median ppFEV_1_ values (85%; IQR: 72%–97.25%) compared to those with recurrent SCV detections (71%; IQR: 56%–91%). This could suggest that poorer lung function may be a risk factor for recurrent SCV detection (z = −2.878; *p* = 0.004). Also, linear regression analysis (Fig. 3) demonstrated a significant negative correlation between the number of SCV detections and the ppFEV_1_ value at the visit preceding the first detection (*p* < 0.00001).

Pre-detection median ppFEV1 values were consistently lower in adults than in children across both groups; however, this difference reached statistical significance only in the single detection group (*p* = 0.040), likely reflecting greater variability in the recurrent group (Supplementary Table F, Supplementary Table G).

At the time of SCV detection, patients with recurrent detections more frequently exhibited co-detection of other bacteria. A statistically significant difference was observed in the frequency of Pa detection between patients with single versus recurrent SCV detections (χ² = 3.938, *p* = 0.049). Chronic Pa was in 41/154 (26.62%, 95% CI: 20.27–34.11%) pwCF and of these, 14/41 (34.15%, 95% CI: 21.56–49.45%) had a single SCV detection, and 27/41 (65.85%, 95% CI: 50.55–78.44%) had recurrent SCV detections.

BMI values did not differ significantly between pwCF with single (Supplementary Table H) and recurrent (Supplementary Table I) SCV detections (*p* = 0.144).

Regarding chronic therapy at the time of SCV detection, oral azithromycin was used by 20/57 (35.09%, 95% CI: 24.00–48.06.00.06%) pwCF with single SCV detections and by 37/57 (64.91%, 95% CI: 51.94–76.00.94.00%) of those with recurrent SCV detections (χ² = 5.505; *p* = 0.020). Inhaled antibiotic use was also more common in pwCF with recurrent detections (62.96%, 34/54, 95% CI: 49.63–74.58%) compared to those with single detection (37.04%, 20/54, 95% CI: 25.42–50.37%), although this difference was not statistically significant. Most patients received at least one antibiotic therapy in the year preceding the first SCV detection. TMP-SMX was used by 58/154 (37.66%, 95% CI: 30.40–45.53.40.53%) pwCF. Specifically, TMP-SMX was used by 19/54 (35.19%) of pwCF with a single SCV detection and by 39/54 (72.22%) of those with recurrent SCV detections (χ² = 8.003; *p* = 0.005).

Univariate logistic regression analysis identified age ≥ 18 years (OR: 2.058, *p* = 0.031), ppFEV_1_ < 80%, (OR: 0.494, *p* = 0.035), chronic Pa colonization (OR: 2.107, *p* = 0.049), chronic oral azithromycin therapy (OR: 2.228, *p* = 0.020), and prior use of TMP-SMX (OR: 2.639, *p* = 0.005) as potential risk factors for recurrent SCV detection.

Multivariate analysis revealed that only prior use of TMP-SMX was an independent risk factor for recurrent SCV detections (OR: 2.280, *p* = 0.028, 95% CI: 1.10–4.74%) (Table 1).

**Table 1:** Univariate and multivariate analyses

				Univariate analysis			Multivariate analysis		
Variable	One SCV detection (*n*/*N*)	Recurrent SCVs detections (*n*/*N*)	Total (*n*/*N*)	OR	95% CI	*P* value	OR	95% CI	*P* value
Age ≥ 18 years	37/73	55/81	92/154	2.058	1.070–3.960	0.031	1.457	0.705–3.010	0.309
ppFEV_1_ < 80%	28/73	40/81	68/154	0.494	0.256–0.952	0.035	0.8265	0.376–1.815	0.635
Oral azithromycin	20/73	37/81	57/154	2.228	1.135–4.377	0.020	1.313	0.604–2.852	0.492
Pa chronic colonisation	14/73	27/81	41/154	2.107	1.002–4.432	0.049	1.695	0.743–3.871	0.210
TMP-SMX	19/73	39/81	54/154	2.639	1.336–5.213	0.005	2.280	1.095–4.745	0.028

SCV: small colony variants; Pa: *pseudomonas aeruginosa*

TMP-SMX: trimethoprim-sulfamethoxazole

ppFEV1: percent predicted forced expiratory volume

## Discussion

This study revealed the presence of SCVs in over a third of a cohort of pwCF and identifies the use of TMP-SMX as a significant risk factor for recurrent SCV detections. We hypothesized that multiple detections might be associated with clinical deterioration, potentially increasing gradually with the number of detections, whereas a single detection might have limited clinical significance. Indeed, patients with more frequent SCV detections exhibited reduced ppFEV_1_ and a decline in respiratory function following the third detection. AST indicated resistance to TMP-SMX in the majority of SCV isolates, with only three exceptions. Finally, a reduction in the number of SCV detections was observed following the initiation of ETI therapy. These are novel findings in the literature.

Previous data by Wolter et al. show a 14−35.3% range of SCV detection [13] among pwCF. The higher SCV detection rate in our cohort compared to Wolter et al.’s pediatric cohort could be attributed to the broader age range (1–65 years) of our study population. Yagci et al. [26], in a monocentric study on a cohort of pwCF aged 1–58 years with a mean age of 9.9 years, reported a lower SCV detection (8.1%), potentially reflecting the younger age of their cohort. Our finding of increased SCV detection in older patients aligns with another monocentric study [27] analyzing pwCF aged 0–61 years. We did not observe significant gender differences between patients with single and recurrent SCV detections.

In our study, regarding potential risk factors (Table 2), prior antibiotic therapy, particularly TMP-SMX use within the year preceding the first SCV detection, emerged as a significant factor. This observation corroborates Ryan et al.’ systematic review [28], which demonstrated a correlation between TMP-SMX use and higher SCV detection rates. Furthermore, in our study, patients with recurrent SCV detections experienced a higher number of antibiotic exposures during the study period compared with those with only a single detection. This association likely reflects the selection pressure exerted by TMP-SMX, which suppresses wild-type *S. aureus* and favors the survival of pre-existing SCVs. However, this association could also reflect the fact that more frequent antibiotic use often indicates more advanced lung disease, which itself might predispose patients to SCV detection. Antibiotic exposure may induce mutations resulting in SCV auxotrophy, impairing the synthesis of essential molecules and reducing cell turnover (hence the term “slow-growing” SCV). This allows SCVs to evade immune clearance, promoting their persistence within host cells and contributing to chronic infections.

**Table 2:** Factors possibly related or not-relate to SCV detection

Related factors	Non-related factors
Chronic colonisation with *P. aeruginosa*	Gender
Older age	BMI
Lower (< 80%) ppFEV_1_	Genotype
Oral azithromycin	Pancreatic sufficiency
Trimethoprim-sulfamethoxazole intake in the previous year	Diabetes

SCVs are characterized by dynamic instability, transitioning between the wild-type and SCV phenotypes in response to environmental pressures. Prolonged exposure to adverse environmental factors can lead to SCV stabilization, resulting in obligate intracellular persistence [14].

Wolter et al. [13] suggested a possible role for chronic therapies, reporting an association between the use of dornase alfa and increased SCV detection risk, potentially due to its mechanism of action: lysing DNA and releasing thymidine, a molecule required by auxotrophic SCVs. Conversely, hypertonic saline was associated with fewer SCV detections. In our study, we did not observe significant differences between pwCF with single versus recurrent SCV detection groups regarding these specific therapies. Inhaled antibiotic therapy was more common in the recurrent colonization group, although this difference was not statistically significant. Oral azithromycin use was significantly more frequent in the recurrent colonization group than in the single detection group in the univariate analysis; however, in the multivariate model, only prior TMP-SMX use remained an independent risk factor for recurrent SCV detection. Colonisation with Pa has been implicated in the transition from wild-type *S. aureus* to the SCV phenotype. Besier et al. [27] reported higher Pa colonisation rates in patients with *S. aureus* SCV compared to those with wild-type *S. aureus*. Consistent with this, our research found that chronic Pa was more frequently retrieved in patients with recurrent detection.

This bacterium may promote SCV emergence through the production of molecules such as 4-hydroxy-2-heptylquinoline-N-oxide, which can protect *S. aureus* from certain antibiotics [29].

Previous studies have reported lower ppFEV1 in pwCF with SCV compared to those without [27, 28], reporting an 18% lower ppFEV_1_ in patients with SCV than in those with wild-type *S. aureus*. Our study found a 14% lower median ppFEV1 in the recurrent SCV detection group compared to the single detection group, suggesting that a poorer baseline clinical condition may be a risk factor for SCV detection. While Wolter et al. [13] also found lower ppFEV_1_ values in subjects with SCV, their analysis suggested that SCV detection did not cause a significant decrease in ppFEV_1_.

Our study reveals that nearly all SCV isolates exhibited resistance to TMP-SMX in AST. This contrasts with Yagci et al. [26], who reported a 16.7% TMP-SMX resistance with no significant difference between SCV and wild-type *S. aureus*. SCVs are often resistant to multiple antibiotics, including sulfonamides [13], rifampicin, moxifloxacin, TMP-SMX [30], and aminoglycosides [31], and exhibit elevated minimum inhibitory concentration (MIC) for linezolid [30]. SCVs exhibited relatively higher vancomycin MICs compared with wild-type S. aureus isolates, and vancomycin exposure may promote SCV selection by suppressing the growth of wild-type strains [30]. Fluoroquinolones and oritavancin may be effective against SCVs [32]. Currently, definitive data establishing optimal treatment for SCV eradication are lacking.

Finally, our study observed a decrease in SCV detections following the introduction of ETI therapy, suggesting a potential association. However, further investigation is required to confirm this trend.

This study is limited by its retrospective and monocentric design, the small number of pediatric pwCF, and the lack of a control group of pwCF who have never had a SCV detection. Because most pwCF had not yet initiated CFTR modulator therapies at the time of their first SCV detection, we were unable to fully assess the impact of these new drugs on SCV detection and clinical implications. However, a decrease in SCV detection rate was observed in the years following the introduction of ETI. Additionally, the data analysis did not distinguish between SCV isolates from throat swabs and sputum samples or between MRSA and MSSA. Furthermore, the shift towards throat swabs following ETI introduction introduces potential bias, and the AST provided only S (sensitive), I (intermediate), or R (resistant) data for SCVs, lacking MIC values. In our study, the total number of cultures performed per subject was not systematically recorded; however, as respiratory cultures were obtained according to a standardized protocol (at least every 3 months and in case of clinical deterioration), the risk of significant sampling bias is likely reduced. Among the limitations, we acknowledge that causality cannot be inferred. While repeated SCV detections coincided with clinical deterioration, patients with more advanced lung disease may be inherently more likely to have SCVs detected—owing to more frequent antibiotic exposure, co-infections, greater healthcare utilization and sampling intensity, and other unmeasured confounders—so SCV detection may be a marker of underlying susceptibility rather than a cause. These mechanisms are not mutually exclusive: advanced disease may predispose patients to SCV detection, and recurrent detections could in turn exacerbate the decline in a vicious manner. This interpretation remains speculative and should be tested in prospective longitudinal studies with standardized sampling. Finally, SCV detection methods are heterogeneous and not standardized across studies [33]. The approach we used—inspection of colony size/morphology on mannitol salt agar—preferentially identifies thymidine-dependent SCVs (often selected by trimethoprim–sulfamethoxazole), whereas other common auxotrophs (e.g., hemin or menadione dependence) may require re-culture on indicator media with specific supplementation and prolonged incubation. Consequently, misclassification and under-ascertainment are possible, and cross-study comparisons are difficult. These methodological constraints could have biased our prevalence estimates and the observed associations. Future work should adopt standardized, multi-step diagnostic algorithms (including auxotrophy testing) to improve sensitivity and comparability.

## Conclusions

In conclusion, our findings suggest that a complex interplay of factors—including older age (likely reflecting longer disease duration), compromised respiratory function, prolonged exposure to antibiotic therapies (particularly TMP-SMX), and chronic Pa colonization—contributes to the frequency of detection of S. aureus SCVs. While SCV detection does not appear to be significantly associated with clinical deterioration at the time of first detection, its role may become more prominent over time, as suggested by the progressive decline in ppFEV_1_ values observed in patients with repeated SCV detections. Similarly, SCV persistence and repeated detection could be associated with increased risk of respiratory exacerbations. Further prospective, multicentric studies, incorporating appropriate control populations, are crucial to thoroughly evaluate the risk factors and clinical impact of SCV in pwCF. Finally, the influence of new CFTR modulator therapies on SCV detection and clinical course warrants further investigation.

## Supplementary Information

Below is the link to the electronic supplementary material.

Supplementary Material 1(DOC 31.5 KB)

Supplementary Material 2(DOC 35.0 KB)

Supplementary Material 3(DOC 29.0 KB)

Supplementary Material 4(DOC 31.5 KB)

Supplementary Material 5(DOC 31.5 KB)

Supplementary Material 6(DOC 128 KB)

Supplementary Material 7(DOC 31.0 KB)

Supplementary Material 8(DOC 34.5 KB)

Supplementary Material 9(DOC 30.0 KB)

## Data Availability

No datasets were generated or analysed during the current study.
