# Supplementary material for: Prevalence and risk factors for recurrent Staphylococcus aureus small-colony variants in people with cystic fibrosis followed at the Tuscan Regional Reference Center
Source: Eur J Clin Microbiol Infect Dis. 2025 Oct 30;45(2):441–9. doi: 10.1007/s10096-025-05313-3 (PMC12987778; doi:10.1007/s10096-025-05313-3)
Supplement: Supplementary file 1 — Supplementary Material 1(DOC 31.5 KB) [file 10096_2025_5313_MOESM1_ESM.doc]

Supplementary Table I. Median and IQR of BMI in patients with multiple detections

| **Variable** | **Median (IQR) (Kg/m^2^)** |
| --- | --- |
| **BMI pre- 1^st^ detection** | 20.01 (18.4–22.46) |
| **BMI at the 1^st^ detection** | 19.78 (18.19–22.18) |
| **BMI post- 1^st^ detection** | 20.03 (18.46–21.99) |
| **BMI al 2^nd^ detection** | 20.36 (18.55–21.94) |
| **BMI al 3^rd^ detection** | 20.63 (18.86–22.58) |
| **BMI at ≥4^th^ detection** | 20.42 (18.63–22.49) |
| *Note: p = 0.144 | |
