# Supplementary material for: Prevalence and risk factors for recurrent Staphylococcus aureus small-colony variants in people with cystic fibrosis followed at the Tuscan Regional Reference Center
Source: Eur J Clin Microbiol Infect Dis. 2025 Oct 30;45(2):441–9. doi: 10.1007/s10096-025-05313-3 (PMC12987778; doi:10.1007/s10096-025-05313-3)
Supplement: Supplementary file 2 — Supplementary Material 2(DOC 35.0 KB) [file 10096_2025_5313_MOESM2_ESM.doc]

Supplementary Table A. Demographic information and clinical data of the study cohort.

| **Demographic data collected** | **Data collected at pre SCV detection visit** | **Data collected at SCV detection visit** | **Data collected at post SCV detection visit** |
| --- | --- | --- | --- |
| Date of birth | ppFEV_1_ | Date of detection | ppFEV_1_ |
| Gender | Weight | Material of detection | Weight |
| Mode of CF diagnosis | BMI | Other pathogens | BMI |
| Age of CF diagnosis | Number of exacerbations in the year before detection | Antibiotic resistance | Number of exacerbations in the year after detection |
| Genotype | Antibiotic administration in the year before detection | ppFEV_1_ | Antibiotic administration in the year after detection |
| Pancreatic sufficiency | TMP-SMX administration in the year before detection | Weight | TMP-SMX administration in the year after detection |
|  | Number of antibiotic cycles in the year before detection | BMI | Number of antibiotic cycles in the year after detection |
|  |  | Symptoms |  |
|  |  | Antibiotic administration |  |
|  |  | Number of antibiotic cycles at the detection visit |  |
|  |  | Ongoing therapy |  |

SCV: small colony variants

CF: cystic fibrosis

ppFEV_1_: percent predicted forced expiratory volume in one second

BMI: body mass index

TMP-SMX: trimethoprim-sulfamethoxazole
