# Supplementary material for: Prevalence and risk factors for recurrent Staphylococcus aureus small-colony variants in people with cystic fibrosis followed at the Tuscan Regional Reference Center
Source: Eur J Clin Microbiol Infect Dis. 2025 Oct 30;45(2):441–9. doi: 10.1007/s10096-025-05313-3 (PMC12987778; doi:10.1007/s10096-025-05313-3)
Supplement: Supplementary file 3 — Supplementary Material 3(DOC 29.0 KB) [file 10096_2025_5313_MOESM3_ESM.doc]

Supplementary Table H. Median and IQR of BMI in patients with a single detection

| **Variable** | **Median (IQR) (Kg/m^2^)** |
| --- | --- |
| **BMI pre- detection** | 19.45 (16.84–22.38)* |
| **BMI at the detection** | 19.61 (17.20–22.24) |
| **BMI post- detection** | 19.68 (17.24–22)* |
| *Note: p = 0.373 | |
