# Supplementary material for: Prevalence and risk factors for recurrent Staphylococcus aureus small-colony variants in people with cystic fibrosis followed at the Tuscan Regional Reference Center
Source: Eur J Clin Microbiol Infect Dis. 2025 Oct 30;45(2):441–9. doi: 10.1007/s10096-025-05313-3 (PMC12987778; doi:10.1007/s10096-025-05313-3)
Supplement: Supplementary file 4 — Supplementary Material 4(DOC 31.5 KB) [file 10096_2025_5313_MOESM4_ESM.doc]

Supplementary Table E. Median and IQR of FEV_1_ in patients with multiple detections

| **Variable** | **Median (IQR) (%)** |
| --- | --- |
| **FEV_1_ pre- 1^st^ detection** | 71 (56–91)* |
| **FEV_1_ at 1^st^ detection** | 71 (55–87) |
| **FEV_1_ post- 1^st^ detection** | 72.5 (55–88) |
| **FEV_1_ at 2^nd^ detection** | 74 (54.5–88) |
| **FEV_1_ at 3^rd^ detection** | 72.5 (55–85.75) |
| **FEV_1_ at ≥4^th^ detection** | 57 (45–79)* |
| *Note: p = 0.025 | |
