# Supplementary material for: Prevalence and risk factors for recurrent Staphylococcus aureus small-colony variants in people with cystic fibrosis followed at the Tuscan Regional Reference Center
Source: Eur J Clin Microbiol Infect Dis. 2025 Oct 30;45(2):441–9. doi: 10.1007/s10096-025-05313-3 (PMC12987778; doi:10.1007/s10096-025-05313-3)
Supplement: Supplementary file 5 — Supplementary Material 5(DOC 31.5 KB) [file 10096_2025_5313_MOESM5_ESM.doc]

Supplementary Table G. Median and IQR of FEV1, divided by patient age in patients with multiple detections

|  | **N° (% of cases)** | **Median (IQR) FEV_1_ pre- 1^st^ detection (%)** | **Median (IQR)**  **FEV_1_ at the**  **1^st^ detection (%)** | **Median (IQR) FEV_1_ post- 1st detection (%)** |
| --- | --- | --- | --- | --- |
| **<18 years** | 26 (32.1%) | 84.5  (61.75–95)^1^ | 81.5  (56.25–90.75)^2^ | 75  (60–91.5)^3^ |
| **≥18 years** | 55 (67.9%) | 70  (55–87.5)^1^ | 67  (55–82.5)^2^ | 69  (55–87)^3^ |
| ^1^Note: p = 0.114  ^2^Note: p = 0.208  ^3^Note: p = 0.453 | | | | |
