# Supplementary material for: Prevalence and risk factors for recurrent Staphylococcus aureus small-colony variants in people with cystic fibrosis followed at the Tuscan Regional Reference Center
Source: Eur J Clin Microbiol Infect Dis. 2025 Oct 30;45(2):441–9. doi: 10.1007/s10096-025-05313-3 (PMC12987778; doi:10.1007/s10096-025-05313-3)
Supplement: Supplementary file 6 — Supplementary Material 6(DOC 128 KB) [file 10096_2025_5313_MOESM6_ESM.doc]

Supplementary Table B. Baseline characteristics of enrolled patients at the time of first detection.

| **Variable** | | | **N (%)**  **Patients colonized only once** | **N (%)**  **Patients colonized more than once** | **N (%)**  **Total patients** | **p** |
| --- | --- | --- | --- | --- | --- | --- |
| Gender | Male | | 48 (65.75%) | 42 (51.85%) | 90 (58.44%) | 0.080 |
|  | Female | | 25 (34.25%) | 39 (48.15%) | 64 (41.56%) |  |
| Diagnosis | Screening | | 40 (54.79%) | 39 (48.15%) | 79 (51.30%) | 0.410 |
|  | Symptoms | | 29 (39.73%) | 36 (44.44%) | 65 (42.21%) | 0.554 |
|  | Familiarity | | 0 (0.00%) | 2 (2.47%) | 2 (1.30%) | 0.498 |
|  | Prenatal | | 0 (0.00%) | 1 (1.23%) | 1 (0.65%) | 1.000 |
|  | Symptoms and familiarity | | 4 (5.48%) | 3 (3.70%) | 7 (4.55%) | 0.709 |
| CFTR genotype | *other^1^*/*other* | | 17 (23.29%) | 18 (22.22%) | 35 (22.73%) | 0.875 |
|  | F508del/*other* | | 39 (53.42%) | 44 (54.32%) | 83 (53.90%) | 0.911 |
|  | F508del/F508del | | 17 (23.29%) | 18 (22.22%) | 35 (22.73%) | 0.875 |
|  | Missing | | 0 (0.00%) | 1 (1.23%) | 1 (0.65%) | 1.000 |
| Pancreatica sufficiency | Yes | | 19 (26.03%) | 8 (9.88%) | 27 (17.53%) | 0.008 |
|  | No | | 54 (73.97%) | 73 (90.12%) | 127 (82.47%) |  |
| Diabetes | Yes | | 10 (13.70%) | 21 (25.93%) | 31 (20.13%) | 0.059 |
|  | No | | 57 (78.08%) | 57 (70.37%) | 114 (74.03%) |  |
|  | Missing | | 6 (8.22%) | 3 (3.70%) | 9 (5.84%) |  |
| Atypical mycobacteriosis | Yes | | 2 (2.74%) | 1 (1.23%) | 3 (1.95%) | 0.604 |
|  | No | | 71 (97.26%) | 80 (98.77%) | 151 (98.05%) |  |
| Other contemporary colonization | *P. aeruginosa chronic colonisation* | | 35 (47.95%)  14 (19.18%) | 54 (66.67%)  27 (33.33%) | 89 (57.79%)  33 (21.43%) | 0.019  0.049 |
|  | No | | 38 (52.05%) | 27 (33.33%) | 65 (42.21%) |  |
| Symptoms at 1st detection | Yes | | 9 (12.33%) | 55 (67.90%) | 35 (22.73%) | <0.00001 |
|  | No | | 64 (87.67%) | 26 (32.10%) | 119 (77.27%) |  |
| Antibiotic at 1st detection | Yes | | 14 (19.18%) | 28 (34.57%) | 42 (27.27%) | 0.032 |
|  | Trimethoprim-sulfamethoxazole  Trimethoprim-sulfamethoxazole +  other  Other | | 2 (2.74%)  4 (5.48%)  8 (10.96%) | 5 (6.17%)  7 (8.64%)  16 (19.75%) | 7 (16.67%)  11 (26.19%)  24 (57.14%) |  |
|  | No | | 59 (80.82%) | 53 (65.43%) | 112 (72.72%) |  |
| Number of exacerbations in previous year | 0 – 1 | | 36 (49.32%) | 17 (20.99%) | 53 (34.42 %) | 0.0002 |
|  | 2 – 4 | | 31 (42.47%) | 40 (49.38%) | 71 (46.10 %) | 0.390 |
|  | 5 – 8 | | 6 (8.22%) | 18 (22.22%) | 24 (15.58 %) | 0.017 |
|  | ≥ 9 | | 0 (0.00%) | 6 (7.41%) | 6 (3.90%) | 0.030 |
| Antibiotic intake in previous year | Yes | | 52 (71.73%) | 73 (90.12%) | 125 (81.17%) | 0.003 |
|  | No | | 21 (28.77%) | 8 (9.88%) | 29 (18.83%) |  |
| Number of oral courses of antibiotic in previous year | 0 – 1 | | 39 (53.42%) | 22 (27.16%) | 61 (39.61%) | 0.0009 |
|  | 2 – 4 | | 30 (41.10%) | 42 (51.85%) | 72 (46.75%) | 0.182 |
|  | 5 – 8 | | 4 (5.48%) | 15 (2.47%) | 19 (12.34%) | 0.015 |
|  | ≥ 9 | | 0 (0.00%) | 2 (27.16%) | 2 (1.30%) | 0.498 |
| Number of intravenous courses of antibiotic in previous year | 0 – 1 | | 69 (94.52%) | 63 (77.78%) | 132 (85.71%) | 0.005 |
|  | 2 – 4 | | 4 (5.48%) | 15 (18.52%) | 19 (12.34%) | 0.015 |
|  | 5 – 8 | | 0 (0.00%) | 3 (3.70%) | 3 (1.95%) | 0.247 |
|  | ≥ 9 | | 0 (0.00%) | 0 (0.00%) | 0 (0.00%) | 1.000 |
| Number of trimetoprim-sulfametossazolo intake in previous year | 0 – 1 | | 67 (91.78%) | 59 (72.84%) | 126 (81.82%) | 0.002 |
|  | 2 – 4 | | 6 (8.22%) | 19 (23.46%) | 25 (16.23%) | 0.010 |
|  | 5 – 8 | | 0 (0.00%) | 3 (3.70%) | 3 (1.95%) | 0.247 |
|  | ≥ 9 | | 0 (0.00%) | 0 (0.00%) | 0 (0.00%) | 1.000 |
| Therapy at the 1st detection | Elexacaftor-ivacaftor-tezacaftor | Yes | 7 (9.59%) | 0 (0.00%) | 7 (4.55%) | 0.005 |
|  |  | No | 66 (90.41%) | 81 (100.00%) | 147 (95.45%) |  |
|  | Ivacaftor | Yes | 9 (12.33%) | 2 (2.47%) | 11 (7.14%) | 0.026 |
|  |  | No | 64 (87.67%) | 79 (97.53%) | 143 (92.86%) |  |
|  | Lumacaftor/ivacaftor | Yes | 7 (9.59%) | 7 (8.64%) | 14 (9.09%) | 0.838 |
|  |  | No | 66 (90.41%) | 74 (91.36%) | 140 (90.91%) |  |
|  | Tezacaftor/ivacafator | Yes | 2 (2.74%) | 0 (0.00%) | 2 (1.30%) | 0.223 |
|  |  | No | 71 (97.26%) | 81 (100.00%) | 152 (98.70%) |  |
|  | Pulmozyme | Yes | 44 (60.27%) | 57 (70.37%) | 101 (65.58%) | 0.188 |
|  |  | No | 29 (39.37%) | 24 (29.63%) | 53 (34.42%) |  |
|  | Creon | Yes | 54 (73.97%) | 73 (90.12%) | 127 (82.47%) | 0.008 |
|  |  | No | 19 (26.03%) | 8 (9.88%) | 27 (17.53%) |  |
|  | Inhaled antibiotics | Yes  Colistin  Tobramycin  Azithromycin  Levofloxacin | 20 (27.40%)  12 (16.44%)  10 (13.70%)  2 (2.74%)  1 (1.37%) | 34 (41.98%)  14 (17.28%)  13 (16.05%)  9 (11.11%)  7 (8.64%) | 54 (35.06%)  26 (38.24%)  23 (33.82%)  11 (16.18%)  8 (11.76%) | 0.058 |
|  |  | No | 53 (72.60%) | 47 (58.02%) | 100 (64.94%) |  |
|  | Bronchodilators | Yes | 56 (76.71%) | 64 (79.01%) | 120 (77.92%) | 0.731 |
|  |  | No | 17 (23.29%) | 17 (20.99%) | 34 (22.08%) |  |
|  | Hypertonic salin solution | Yes | 9 (12.33%) | 13 (16.05%) | 22 (14.29%) | 0.510 |
|  |  | No | 64 (87.67%) | 68 (83.95%) | 132 (85.71%) |  |
|  | Oral azithromycin | Yes | 20 (27.40%) | 37 (45.68%) | 57 (37.01%) | 0.020 |
|  |  | No | 53 (72.60%) | 44 (54.32%) | 97 (62.99%) |  |
|  | Inhaled corticosteroids | Yes | 43 (58.90%) | 44 (54.32%) | 87 (56.49%) | 0.567 |
|  |  | No | 30 (41.10%) | 37 (45.68%) | 67 (43.51%) |  |
|  | Respiratory physiotherapy | Yes | 68 (93.15%) | 80 (98.77%) | 148 (96.10%) | 0.102 |
|  |  | No | 5 (6.85%) | 1 (1.23%) | 6 (3.90%) |  |
| Classes of ppFEV_1_  before the 1st detection | < 40 | | 5 (6.85%) | 8 (9.88%) | 13 (8.44%) | 0.571 |
|  | 40-79 | | 23 (31.51%) | 40 (49.38%) | 63 (40.91%) | 0.024 |
|  | ≥ 80 | | 39 (53.42%) | 33 (40.74%) | 72 (46.75%) | 0.115 |
|  | Missing^2^ | | 6 (8.22%) | 0 (0.00%) | 6 (3.90%) |  |
| Classes of BMI  before the 1st detection | < 18 | | 24 (32.88%) | 18 (22.22%) | 42 (27.27%) | 0.138 |
|  | 18 – 24.9 | | 41 (56.16%) | 55 (67.90%) | 96 (62.34%) | 0.133 |
|  | 25 – 29.9 | | 6 (8.22%) | 8 (9.88%) | 14 (9.09%) | 0.721 |
|  | ≥ 30 | | 1 (1.37%) | 0 (0.00%) | 1 (0.65%) | 0.474 |
|  | Missing | | 1 (1.37%) | 0 (0.00%) | 1 (0.65%) |  |
| ^1^*other*: different variant from F508del  ^2^patients under the age of 6, who were unable to undergo spirometry | | | | | |  |
