# Supplementary material for: Prevalence and risk factors for recurrent Staphylococcus aureus small-colony variants in people with cystic fibrosis followed at the Tuscan Regional Reference Center
Source: Eur J Clin Microbiol Infect Dis. 2025 Oct 30;45(2):441–9. doi: 10.1007/s10096-025-05313-3 (PMC12987778; doi:10.1007/s10096-025-05313-3)
Supplement: Supplementary file 7 — Supplementary Material 7(DOC 31.0 KB) [file 10096_2025_5313_MOESM7_ESM.doc]

Supplementary Table F. Median and IQR of FEV_1_, divided by patient age in patients with a single detection

|  | **N° (% of cases)** | **Median (IQR) FEV_1_**  **pre- detection (%)** | **Median (IQR) FEV_1_ at the detection (%)** | **Median (IQR)**  **FEV_1_**  **post- detection (%)** |
| --- | --- | --- | --- | --- |
| **<18 years** | 36 (49.32%) | 90.5  (81–101)^1^ | 92  (78.5–100)^2^ | 92.5  (74–101.00)^3^ |
| **≥18 years** | 37 (50.68%) | 79  (71–90)^1^ | 78  (61.25–87.25)^2^ | 80  (66–87.5)^3^ |
| ^1^Note: p = 0.040  ^2^Note: p = 0.024  ^3^Note: p = 0.027 | | | | |
