# Supplementary material for: Prevalence and risk factors for recurrent Staphylococcus aureus small-colony variants in people with cystic fibrosis followed at the Tuscan Regional Reference Center
Source: Eur J Clin Microbiol Infect Dis. 2025 Oct 30;45(2):441–9. doi: 10.1007/s10096-025-05313-3 (PMC12987778; doi:10.1007/s10096-025-05313-3)
Supplement: Supplementary file 8 — Supplementary Material 8(DOC 34.5 KB) [file 10096_2025_5313_MOESM8_ESM.doc]

Supplementary Table C. Number of detections over the years, divided by patient age

| **Year** | **<18 years**  **Number of colonization (row total percentage)** | **>18 years**  **Number of colonization (row total percentage)** | **Total**  **Number of colonization (column total percentage)** |
| --- | --- | --- | --- |
| **2017** | 25 (29.41%) | 60 (70.59%) | 85 (19.06%) |
| **2018** | 48 (32.87%) | 97 (67.13%) | 146 (32.51%) |
| **2019** | 22 (34.38%) | 43 (65.62%) | 64 (14.57%) |
| **2020** | 8 (24.24%) | 25 (75.76%) | 33 (7.40%) |
| **2021** | 20 (35.71%) | 36 (64.29%) | 56 (12.56%) |
| **2022** | 11 (30.56%) | 25 (69.44%) | 36 (8.07%) |
| **2023** | 12 (46.15%) | 14 (53.85%) | 26 (5.83%) |
| **Total** | 146 (32.74%) | 300 (67.26%) | 446 (100.00%) |
