# Supplementary material for: Prevalence and risk factors for recurrent Staphylococcus aureus small-colony variants in people with cystic fibrosis followed at the Tuscan Regional Reference Center
Source: Eur J Clin Microbiol Infect Dis. 2025 Oct 30;45(2):441–9. doi: 10.1007/s10096-025-05313-3 (PMC12987778; doi:10.1007/s10096-025-05313-3)
Supplement: Supplementary file 9 — Supplementary Material 9(DOC 30.0 KB) [file 10096_2025_5313_MOESM9_ESM.doc]

Supplementary Table D. Number of detections after starting CFTR modulators.

| Year | Number of detections | Number of detections in pwCF using ETI | Number of pwCF using ETI | Number of detections in pwCF using Ivacaftor | Number of detections in pwCF using Lumacaftor/ivacaftor | Number of detections in pwCF using Tezacaftor/ivacafator |
| --- | --- | --- | --- | --- | --- | --- |
| 2017 | 85 | Not approved | Not approved | 2 | 4 | 0 |
| 2018 | 145 | Not approved | Not approved | 2 | 13 | 0 |
| 2019 | 65 | 2 | 12 | 3 | 10 | 0 |
| 2020 | 33 | 4 | 9 | 4 | 5 | 0 |
| 2021 | 56 | 8 | 13 | 7 | 7 | 0 |
| 2022 | 36 | 15 | 12 | 15 | 0 | 3 |
| 2023 | 26 | 9 | 9 | 11 | 0 | 1 |
